# The prospective impact of food pricing on improving dietary consumption: A systematic review and meta-analysis

**DOI:** 10.1371/journal.pone.0172277

**Published:** 2017-03-01

**Authors:** Ashkan Afshin, José L. Peñalvo, Liana Del Gobbo, Jose Silva, Melody Michaelson, Martin O'Flaherty, Simon Capewell, Donna Spiegelman, Goodarz Danaei, Dariush Mozaffarian

**Affiliations:** 1 Institute for Health Metrics and Evaluation, University of Washington, Seattle, WA, United States of America; 2 Friedman School of Nutrition Science & Policy, Tufts University, Boston, MA, United States of America; 3 School of Medicine, Stanford University, Stanford, CA, United States of America; 4 Department of Cardiology, Boston Medical Center, Boston, MA, United States of America; 5 School of Medicine, Tufts University, Boston MA, United States of America; 6 Department of Public Health and Policy, University of Liverpool, Liverpool, United Kingdom; 7 Department of Biostatistics, Harvard T.H. Chan School of Public Health, Boston, MA, United States of America; 8 Department of Epidemiology, Harvard T.H. Chan School of Public Health, Boston, MA, United States of America; 9 Department of Global Health and Population, Harvard T.H. Chan School of Public Health, Boston, MA, United States of America; 10 Department of Nutrition, Harvard T.H. Chan School of Public Health, Boston, MA, United States of America; University of Cambridge, UNITED KINGDOM

## Abstract

**Background:**

While food pricing is a promising strategy to improve diet, the prospective impact of food pricing on diet has not been systematically quantified.

**Objective:**

To quantify the prospective effect of changes in food prices on dietary consumption.

**Design:**

We systematically searched online databases for interventional or prospective observational studies of price change and diet; we also searched for studies evaluating adiposity as a secondary outcome. Studies were excluded if price data were collected before 1990. Data were extracted independently and in duplicate. Findings were pooled using DerSimonian-Laird's random effects model. Pre-specified sources of heterogeneity were analyzed using meta-regression; and potential for publication bias, by funnel plots, Begg's and Egger's tests.

**Results:**

From 3,163 identified abstracts, 23 interventional studies and 7 prospective cohorts with 37 intervention arms met inclusion criteria. In pooled analyses, a 10% decrease in price (i.e., subsidy) increased consumption of healthful foods by 12% (95%CI = 10–15%; N = 22 studies/intervention arms) whereas a 10% increase price (i.e. tax) decreased consumption of unhealthful foods by 6% (95%CI = 4–8%; N = 15). By food group, subsidies increased intake of fruits and vegetables by 14% (95%CI = 11–17%; N = 9); and other healthful foods, by 16% (95%CI = 10–23%; N = 10); without significant effects on more healthful beverages (-3%; 95%CI = -16-11%; N = 3). Each 10% price increase reduced sugar-sweetened beverage intake by 7% (95%CI = 3–10%; N = 5); fast foods, by 3% (95%CI = 1–5%; N = 3); and other unhealthful foods, by 9% (95%CI = 6–12%; N = 3). Changes in price of fruits and vegetables reduced body mass index (-0.04 kg/m^2^ per 10% price decrease, 95%CI = -0.08–0 kg/m^2^; N = 4); price changes for sugar-sweetened beverages or fast foods did not significantly alter body mass index, based on 4 studies. Meta-regression identified direction of price change (tax vs. subsidy), number of intervention components, intervention duration, and study quality score as significant sources of heterogeneity (P-heterogeneity<0.05 each). Evidence for publication bias was not observed.

**Conclusions:**

These prospective results, largely from interventional studies, support efficacy of subsidies to increase consumption of healthful foods; and taxation to reduce intake of unhealthful beverages and foods. Use of subsidies and combined multicomponent interventions appear most effective.

## Introduction

Poor diets are the leading risk factor for mortality and morbidity globally.[1, 2] The World Health Organization and the United Nations General Assembly have called for adoption and implementation of evidence-based government policies to improve diet.[3–6] Whereas fiscal measures such as taxation and subsidies have been proposed as effective strategies,[3–6] most prior evidence of their efficacy for changing diet is derived from cross-sectional modeling studies.[7–9] Such studies provide important information on potential effects of fiscal policies, but may have more limited ability to draw conclusions about the prospective effect of actual price changes on actual changes in consumption. In addition, such studies do not allow assessment of differences in efficacy for price increases (taxation) vs price decreases (subsidies); nor the extent to which other accompanying policy strategies, such as changes in the availability of options or advertising/promotion of price changes, might modify effectiveness. Several reviews suggest that price changes may prospectively improve diet and obesity;[9–14] yet, this evidence has been summarized only qualitatively, without quantitative assessment of effectiveness or key potential sources of heterogeneity. To address these key gaps in knowledge, we systematically investigated and quantified the prospective, empirical effects of change in food price on dietary consumption, and how key additional interventions might modify these effects.

## Methods

We followed the recommendations of the Meta-analysis of Observational Studies in Epidemiology (MOOSE)[15] and of Preferred Reporting Items for Systematic reviews and Meta-Analyses (PRISMA)[16] guidelines in all stages of the design, implementation, and reporting of this meta-analysis (S1 File). The study objective, search strategy, and selection criteria were specified in advance in the Study Protocol (S2 File).

### Primary exposures and outcomes

The primary intervention/exposure of interest was the change in the price of foods or beverages due to taxation, subsides, or other factors. We included studies of multicomponent interventions if studies reported the effect of the price change separately or if the price change was a major component of the intervention. The primary outcome was the change in consumption of foods and beverages; data on sales/purchase were considered a proxy for consumption. Secondary study outcomes included change in body weight and body mass index (BMI).

### Search strategy

We searched multiple online databases in June 2014 including PubMed, Econlit, Embase, Ovid, Cochrane Library, Web of Science, and CINAHL. Search terms were compiled in 3 categories: setting queries (e.g., national, state, city, workplace, schools, supermarket, restaurant, fast food, and cafeteria), intervention queries (e.g., tax, subsidy, incentive, and price) and outcome queries (e.g., food, beverage, fruit, vegetable, soda, meat, dairy, overweight, obesity, and adiposity). The complete list of the search terms and date of search for each database are provided in S3 File. Furthermore, for each of the articles included in the final analysis as well as the relevant reviews identified through search of databases, we hand-searched the reference list and the first 20 “related articles” in PubMed.

### Study selection

We included all interventional (randomized or nonrandomized) and observational (prospective cohort) studies that (a) assessed the relationship between change in food price and change in dietary consumption or adiposity among generally healthy individuals (children or adults); (b) reported the estimated change in the price; and (c) provided an estimate of the change in dietary consumption or adiposity and a measure of uncertainty for the reported change.

We excluded modelling studies, cross-sectional studies, and laboratory experiments (hypothetical situations). Studies were also excluded if (a) all price data were collected before 1990, due to the potential changes in the relation between food prices and consumption over time; (b) outcomes did not include diet or adiposity; or (c) for observational studies, only crude (not multivariable adjusted) effect measures were reported.

### Data extraction

Using a standardized electronic format, 2 investigators extracted data independently and in duplicate on first author name, publication year, study location, design, population (age, sex, race, sample size), duration of follow-up, price data, outcome data (definition, ascertainment methods, change), and (for observational studies) covariates. In addition, 2 investigators independently assessed the quality of studies based on 5 criteria: study design, assessment of exposure, assessment of outcome, control for confounding, and evidence of selection bias (**S1 Table**). For each criterion, each study received a score of 1 or 0 (1 being better), and an overall quality score was calculated as the sum of individual scores. Differences in data extraction and quality assessment between investigators were infrequent and were resolved by consensus.

### Statistical analysis

The primary outcome was the percent change in consumption of foods/beverages due to the percent change in their price. We evaluated both the overall effect of subsidies on healthful items and taxes on unhealthful items; and the effects according to key food groups (e.g. fruits and vegetables). For pooling, each study-specific effect was standardized to a 10% price change, assuming a linear dose-response relationship. Absolute consumption or absolute price changes were not combined due to heterogeneity in currencies, base prices, and base consumptions. Studies only reporting absolute price changes (45, 46, 56, 57, 58, 60), without required information to calculate percentage change, were not included in the quantitative evidence synthesis. The variance of percent change in consumption was calculated based on the variance of the outcome at baseline and end-follow up, assuming a correlation between these measures of 0.5 (S4 File). Study-specific effect sizes were pooled using inverse-variance-weighted random-effect models (metan command in Stata). Cochran's Q and the I^2^ were used to assess the between-study heterogeneity; with I^2^ values of 25%, 50%, and 75% representing low, moderate, and high heterogeneity.[17] Meta-regression (metareg command in Stata) was used to explore potential sources of heterogeneity including design (randomized intervention, nonrandomized intervention, observational), location (US, other), intervention duration (binary, at median), setting (e.g., cafeteria, communities, supermarket, vending machine), population (adults, children, both), direction of price change (increase, decrease), number of additional interventional components (none, 1, 2), type of additional intervention components (none, various types such as changes in availability, promotion/advertising of price change, labeling, nutrition education), and quality score (0–3, 4–5). Publication bias was assessed by visual inspection of funnel plots, Egger's test, and Begg's test. [18] All analyses were conducted with Stata 13.0 software (StataCorp).

To evaluate the strength of the evidence, we assessed 3 different established evidence grading frameworks, including from American Heart Association (AHA),[19] U.S. Preventive Services Task Force (USPSTS),[20] and Centers for Disease Control and Prevention (CDC) Community Guide.[21, 22] **S2 Table** provides a detailed description of each of these grading criteria.

## Results

### Study characteristics

**Fig 1:**
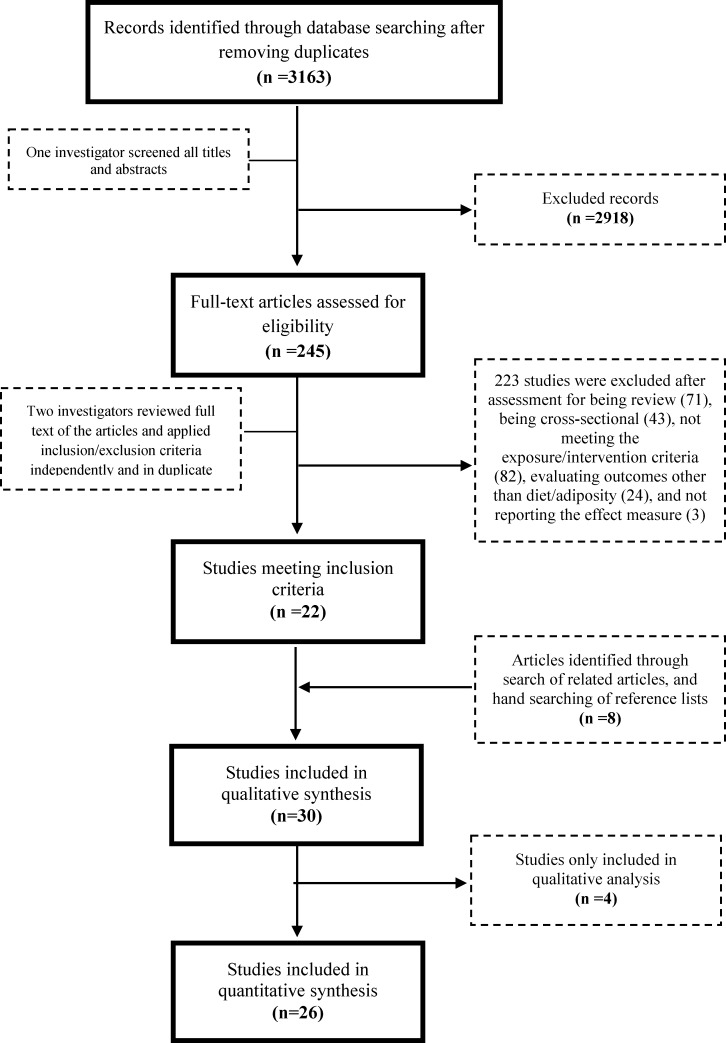
Screening and selection process of interventional trials and prospective observational studies evaluating the relationship between changes in food prices and dietary consumption or adiposity.

Of 3,163 identified articles, 30 met inclusion criteria (**Fig 1**). These included 23 interventional studies (7 randomized, 16 nonrandomized) and 7 prospective cohort studies (**Table 1 and Table 2**).

**Table 1:** Characteristics of the identified studies evaluating the relationship between price change and dietary consumption or adiposity.

Study	Design	Location	Setting	Population	Age Group	Quality Score
An (2013)[23]	Nonrandomized intervention^1^	South Africa	Supermarket^2^	Members of the Discovery health insurance	Adults	4
Anderson (2001)^3^[24]	RCT	US	Farmers' market	Participants in WIC and Community Action Agency Commodity Supplemental Food Program in Genesee County, Michigan	Adults	3
Anliker (1992)^3^[25]	Nonrandomized intervention^4^	US	Farmers' market	Participants in WIC program in Connecticut	Adults	3
Bihan (2012)^3^[26]	RCT	France	Community	Individuals undergoing health examinations at a center affiliated with the French National Insurance System (Social Security)	Adults	3
Blakely (2011)[27]	RCT	New Zealand	Supermarket	Regular supermarket shoppers	Adults	3
Block (2010)[28]	Nonrandomized intervention^4^	US	Cafeteria	Regular cafeteria customers (staff, patients, and visitors at a hospital in Boston)	Adults	5
Brown (2009)[29]	Nonrandomized intervention^1^	US	Vending machine	Statewide representation of Mississippi school students (K-12)	Children	3
Duffey (2010)[30]	Prospective cohort	US	Community	Black and white young adults in the US participating CARDIA study (ages 18–30)	Adults	4
Elbel (2013)[31]	Nonrandomized intervention^1^	US	Cafeteria	Regular consumer in a corner store of a hospital in New York (mostly low-income, minority, and immigrant populations)	Adults	4
Fletcher (2010)[32]	Nonrandomized intervention^1^	US	State	Random sample of state residents	Adults	3
French (1997)[33]	Nonrandomized intervention^1^	US	Vending machine	Regular customers of vending machines in a university	Adults	3
French (1997)[34]	Nonrandomized intervention^1^	US	Cafeteria	Students in 2 US high schools	Children	3
French (2001)[35]	RCT	US	Vending machine	Regular consumer of vending machines (students and workers)	Children/ Adults	3
French (2010)[36]	RCT	US	Vending machine	Regular consumer of vending machines (garage employees and drivers)	Adults	2
Gordon-Larsen (2011)[37]	Prospective cohort	US	Community	A representative sample of US adolescents (grades7–12)	Children	4
Herman (2008)^3^[38]	Nonrandomized intervention^4^	US	Community	Women who enrolled in WIC (post-partum services)	Adults	3
Horgen (2002)[39]	Nonrandomized intervention^1^	US	Restaurant	Regular customers of a restaurant in a relatively affluent urban area	Adults	3
Jeffery (1994)[40]	Nonrandomized intervention^1^	US	Cafeteria	Regular customers of a cafeteria at a university office building	Adults	3
Jue (2012)[41]	Nonrandomized intervention^1^	US	Cafeteria	Regular customers of 3 hospital cafeterias in Philadelphia, PA; Detroit, MI; and Evanston, IL	Adults	3
Khan (2012)[42]	Prospective cohort	US	Community	US children participating in the Early Childhood Longitudinal Study, Kindergarten Class of 1998–99 (ECLS-K).	Children	4
Kocken (2012)[43]	RCT	Netherlands	Vending machine	Regular customers of vending machines in participating schools (students)	Children	3
Kottke (2013)[44]	Nonrandomized intervention^1^	US	Cafeteria	Regular cafeteria customers	Adults	3
Lowe (2010)[45]	Nonrandomized intervention^1^	US	Cafeteria	Regular customers of 2 hospital cafeterias in Philadelphia	Adults	3
Meyer (2014)[46]	Prospective cohort	US	Community	CARDIA participants	Adults	4
Michels (2008)[47]	Nonrandomized intervention^1^	US	Cafeteria	Regular cafeteria customers (students, faculty and staff)	Adults	3
Paine-Andrews (1996)[48]	Nonrandomized intervention^1^	US	Supermarket	Regular supermarket shoppers	Adults	3
Powell (2009)[49]	Prospective cohort	US	Community	US children and mothers participating in National Longitudinal Survey of Youth (NLSY97)	Children	4
Powell (2011)[50]	Prospective cohort	US	Community	Men & Women from PSID study	Adults	4
Waterlander (2013)[51]	RCT	Netherlands	Supermarket	Regular supermarket shoppers	Adults	3
Wendt (2011)[52]	Prospective cohort	US	Community	Participants in Early Childhood Longitudinal Study, Kindergarten Class of 1998–99 (ECLS-K)	Children	4

^1^Nonrandomized intervention without external control group.

^2^Nation-wide studies conducted in 9 provinces of South Africa.

^3^Only included in qualitative review of evidence.

^4^Nonrandomized intervention with external control group

RCT: Randomized controlled trials.

**Table 2:** Characteristics of the intervention (or exposure) and outcome in studies evaluating the relationship between price change and dietary consumption or adiposity.

Study	Targeted Foods/beverages	Type of Price Change	Other Components of Intervention	Price Data Source	Duration of Price Change (Months)	Outcome	Outcome Ascertainment
An (2013)[23]	Healthy foods	Cash-back rebate (10%-25%)	Point of purchase promotion	Scanner sales data and participants credit cards	11	Fruits and vegetables, BMI	Questionnaire
Anderson (2001)[24]	Fruits and vegetables	Coupons ($20)	Nutrition education	Assigned by investigators^1^	2	Fruits and vegetables	Questionnaire
Anliker (1992)[25]	Fruits and vegetables	Coupons ($10)	None	Assigned by investigators	2	Fruits and vegetables	Interview
Bihan (2012)[26]	Fruits and vegetables	Vouchers (10 Euros/Person/Month)	Dietary advice	Assigned by investigators	3	Fruits and vegetables	FFQ
Blakely (2011)[27]	Healthy foods	Discount (12.5%)	Nutrition education	Scanner sales data and personalized scannable card	6	Healthy food, Fruits and vegetables	Scanner sales data and personalized scannable card
Block (2010)[28]	SSBs	Price increase (35%)	Nutrition education	Cash register records	1	SSBs	Cash register records
Brown (2009)[29]	SSBs, fruit juice, sports drink water	Price increase (10%-25%)	Changes in availability, nutrition education	Standardized data collection sheet completed by each participating school	9	SSBs, fruit juice, sports drink, water	Standardized data collection sheet
Duffey (2010)[30]	SSBs, whole milk, burger, pizza	Price increase (10%)	None	C2ER	240	SSBs, whole milk, burger, pizza	Diet history
Elbel (2013)[31]	Less healthy foods and beverages	Price increase (30%)	Labelling, nutrition education	Assigned by investigators	0.3	Less healthy foods	Sales records
Fletcher (2010)[32]	SSBs	Price increase (10%)	None	The Book of the State		BMI	Behavioral Risk Factor Surveillance System
French (1997)[33]	Low-fat products	Discount (50%)	Labelling	Assigned by investigators	0.75	Low-fat products	Sales records
French (1997)[34]	Fruits, carrots, salads	Discount (50%)	Point of purchase promotion	Assigned by investigators	0.75	Fruits, carrots, salads	Sales records
French (2001) [35]	Low-fat products	Discount (10%-50%)	Labeling, promotion		1	Low-fat products	Manual inventory counts
French(2010)[36]	Healthy foods	Discount (10%)	Increased availability by 50%, labeling, other	Sales data from vending machine company	18	Fruits and vegetables, SSBs, snacks/sweets, fast food meals, total energy intake, BMI, weight	FFQ Objectively measured
Gordon-Larsen[37] (2011)	SSBs, burger	Price increase (20%)	None	C2ER	48	SSBs, burgers	Questionnaire
Herman (2008)		Vouchers ($10/Person/week)	None	Assigned by investigators	6	Fruits and vegetables	Interviews with trained nutritionists
Horgen (2002)[39]	Healthy foods	Discount (20%-30%)	Promotion of price reduction	Assigned by investigators	0.75	Chicken sandwich, chicken salad, soup	Electronic sales records
Jeffery (1994)[40]	Fruits, salads	Discount (50%)	Changes in availability	Cash register records	0.75	Fruits, salad	Cash register records
Jue (2012)[41]	Zero-calorie beverages	Discount (10%)	Promotion of price reduction	Cash register records	1.5	Zero-calorie beverages	Cash register records
Khan (2012)[42]	Fast food	Price increase (10%)		ACCRA		Fast food	Self-reported
Kocken (2012)[43]	Lower-calorie products	Discount (10%)	None	Assigned by investigators	1.5	Healthy food, Healthy beverages	Vending machine data
Kottke (2013)[44]	Salad bar	Discount (50%)	None	Cash register records	1	Salad bar	Cash register records
Lowe (2010)[45]	Calories dense food	Discount (15–25%)	changes in availability, nutrition education	Assigned by investigators	3	Calorie	Cash register data and subject's ID card
Meyer (2014)[46]	Fast food	Price increase (22.5)		C2ER		Fast food	Diet history
Michels (2008)[47]	Healthy foods	Discount (20%)	Nutrition education	Cash register records	1.25	Healthy food	Cash register records
Paine-Andrews (1996)[48]	Low fat milk, dressing, and dessert	Discount (20%-25%)	Promoting and product sampling	Assigned by investigators	0.03	Low fat milk, low fat dressing	Trained observers
Powell (2009)[49]	Fruits and vegetables	Price increase (10%)	None	ACCRA	48	BMI	Self-reported anthropometric information
Powell (2011)[50]	Fruits and vegetables, fast food	Price increase ($1)	None	ACCRA	72	BMI	Self-reported anthropometric information
Waterlander (2013)[51]	Fruits and vegetables	Discount (50%)	Nutrition education	Assigned by investigators	6	Fruits and vegetables	Supermarket register receipts
Wendt (2011)[52]	SSBs, Vegetables	Price increase (10%)	None	Food–at–Home Price Database		BMI	Objectively measured

^1^The investigators defined the price changes as part of the intervention.

ACCRA: American Chambers of Commerce Researchers Association; C2ER: Council for Community and Economic Research; FFQ: Food frequency questionnaire.

Studies not providing sufficient information to quantify the magnitude of the price change were only included in qualitative assessment of the evidence (45, 46, 56, 57, 58, 60). Among these, three interventional studies were conducted in the context of the WIC Farmers' Market Nutrition Program (FMNP), in Michigan (56), Connecticut (57), and California (60). Overall, these trials agreed on the direct impact that access to Farmers' Market, and specifically the distribution of coupons, had on increasing frequency of consumption of fresh fruits and vegetables. In the shorter (two months) duration studies this impact was maximized with the combination of a educational interventions (56), or the impact was observed to be only significant among those participants using their food stamps in addition to the provided coupons (57). A six months intervention among women enrolled for postpartum services at WIC sites in Los Angeles (60) those distributed with vouchers showed and increment in their consumption of fruits and vegetables not only after the intervention but also after additional six months of follow up with no intervention (60). The study of Bihan et al (58) focused on low-income population in France and showed increments on the consumption of fruits and vegetables after a short-term (3 months) intervention with either dietary advice alone or in combination with vouchers. Observational studies showed a limited role in weight outcomes of US adults (46), and significant impact was only seen among specific subgroups. Higher prices of fruits and vegetables are related to higher BMI among lower income women, and women with children. Similarly, these observational papers found a modest but measurable impact of fiscal food pricing policies on consumption of fruits, vegetables and fast-food as well as weight outcomes of children 6–17 (59).

Eleven studies assessed the effect of price increases; and 19, of price decreases (subsidies); several of these studies had multiple intervention arms. Study populations included children (N = 7 studies), adults (N = 22), or both (N = 1); and countries included the US (n = 25), The Netherlands (n = 2), New Zealand (n = 1), South Africa (n = 1) and France (n = 1). Price change interventions were conducted in different settings including cafeterias (n = 8), vending machines (n = 5), and supermarkets (n = 4).

The magnitudes of price changes in interventional studies varied from 10% to 50% across studies. In some trials, interventions included other components, such as promotion/advertising of price change, nutrition education, labeling, and changes in availability. Duration of follow-up also varied, with longest follow-up of 18 months in trials [36] and 20 years in prospective cohort studies.[30]

Sugar-sweetened beverages (SSBs) and fast foods were the most common dietary targets for price increases. Target foods in studies of price decreases (subsidies) included fruits, vegetables, salads, and low-fat products. In most studies, the changes in diet were assessed based on objective sales records.

### Effects of price decrease

**Fig 2:**
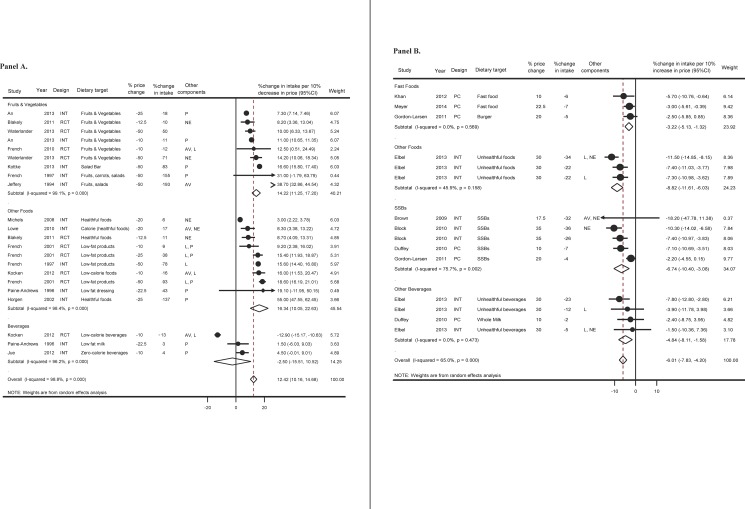
**Prospective relationship of price decrease (Panel A) and increase (Panel B) with dietary consumption. Studies included randomized controlled trials (RCTs), nonrandomized interventions (INT), and prospective cohorts (PC).** Some studies included other intervention components such as advertising/promotion of price change (P), nutrition education (NE), labeling (L), or change in food/beverage availability (AV). Effect sizes were pooled using inverse-variance-weighted random-effect meta-analysis. Statistically significant heterogeneity was seen for all I^2^ values>90% (Q-test p<0.001) and I^2^ = 75% (Q-test p = 0.002), but not I^2^ = 45% (Q-test p = 0.158) or I^2^ = 0% (Q-test p> = 0.470).

Twenty-two intervention studies/arms assessed effects of price decreases (generally in the form of discount at the point of purchase, coupon, or cash rebate) on more healthful foods. Pooling all studies, each 10% decrease in price increased consumption of healthful foods by 12% (95%CI: 10% to 15%) (**Fig 2A**). Fruits and vegetables were the most common target, including studies among adults in the US,[36, 40, 44] New Zealand,[27] South Africa,[23] and The Netherlands[51]; and among children in the US [34]. Most individual studies found significant effects; and pooling all studies, each 10% price decrease increased consumption of fruits and vegetables by 14% (95%CI: 11% to 17%).

Studies evaluating price decreases on other healthful foods (e.g., defined based on lower calorie or fat content) were conducted among adults in the US[33, 35, 39, 45, 47, 48] and New Zealand[27]; among children in the Netherlands[43]; and among both adults and children. As with fruits and vegetables, most individual studies found a significant effect. Pooling all studies, each 10% decrease in the price increased consumption by 16% (95% CI: 10% to 23%) (Fig 2A).

Only 3 interventional trials assessed effects of price decreases on consumption of specific beverages (e.g., low-fat milk, zero-calorie beverages).[41, 43, 48] No significant effect was found in each study or pooling across the 3 studies (Fig 2A).

### Effects of price increase

Fifteen studies/intervention arms assessed the effects of price increases on consumption of unhealthful foods/beverages. These studies included a mix of nonrandomized interventions and prospective cohort studies; all were from the US and included studies conducted among adults [28, 53] and children.[29, 37]. Pooling all studies, each 10% increase in price decreased consumption by 6% (95%CI: 4% to 8%) (Fig 2B). Evaluating food types separately, significant reductions were seen for fast foods, other unhealthful foods, SSBs, and other unhealthful beverages.

### Effects of food pricing on adiposity

One nonrandomized intervention in South Africa and 3 prospective cohort studies in the US evaluated how changes in pricing of specific foods relate to adiposity. The trial evaluated a 10% decrease in the price of fruits and vegetables, implemented as cash-back rebate, over 11 months;[23] and the observational studies, the longitudinal price changes of fruits and vegetables and adiposity. Pooling all 4 studies, each 10% decrease in price of fruits and vegetables was associated with 0.04 kg/m^2^ (95% CI: 0 to 0.08) lower BMI (**S1 Fig**).

Two prospective cohorts assessed the relationship between change in the price of fast foods and BMI among US children[49] and adults;[50] and one nonrandomized intervention and one prospective cohort assessed the relationship between price increase and consumption of sugar-sweetened beverages among US adults[32] and children.[52] Pooling all studies, a nonsignificant trend toward lower BMI was seen, with magnitude similar to the difference in BMI seen in studies of price decreases (per 10% price increase: -0.06 kg/m^2^ (95% CI: -0.16 to 0.03) (S1 Fig).

### Evaluation of heterogeneity

In univariate meta-regression, findings were not significantly different according to differences in study design (randomized intervention, nonrandomized intervention, prospective cohort), location (US, other), setting (cafeteria, community, supermarket, vending machine) duration (months), population (adults, children, both), number of additional intervention components (none, 1–2) type of additional intervention component (none, change in food availability, labeling, nutrition education, food promotion) (P>0.05 each; S3 Table). Statistically significant larger effects were identified in studies with price decreases (subsidies) vs. increases (taxes) (P-heterogeneity = 0.044); and with lower (2–3) vs. higher (4–5) study quality score (P-heterogeneity = 0.034). In multivariate meta-regression including direction of price change and study quality score simultaneously, neither was statistically significant due to collinearity.

### Publication bias

Visual inspection of funnel plots provided mixed evidence for publication bias (**S2 Fig**). However, Begg’s or Eggers test did not identify statistical evidence for publication bias, although numbers of studies in some of these analyses were limited.

### Grading of the evidence

We formally evaluated the evidence from prospective interventional and observational studies for effectiveness of subsidies to improve diet. We found consistent evidence, in direction and size of the effect, from multiple (5) well-designed and executed interventional (randomized or nonrandomized) studies that subsidies were effective in increasing consumption of fruits and vegetables and other healthful foods (**Table 3**). This evidence was found to be consistent with class I A AHA recommendations, Grade A USPSTF recommendations, and “Strong Evidence, Strongly Recommend” CDC Community Guide recommendations. We found consistent evidence, in direction and size of the effect, from fewer (2) well-designed and executed nonrandomized interventions and 1 prospective cohort that taxation reduced the intake of SSBs. This evidence was consistent with class II A AHA recommendations, Grade B USPSTF USPA recommendations, and “Sufficient Evidence, Recommend” CDC Community Guide recommendations. The strength of evidence for effectiveness of subsidies to reduce BMI and taxation to reduce consumption of unhealthful foods or BMI was less robust.

**Table 3:** Results of grading of the prospective interventional and observational evidence for effectiveness of food pricing interventions to improve diet and adiposity.

Policy	American Heart Association^1^	U.S. Preventive Services Task Force^2^	CDC Community Guide^3^
Subsidies			
To increase consumption of fruits and vegetables	Class I, Level of Evidence A	Grade A, High Level of Certainty	Strong Evidence, Strongly Recommended
To increase consumption of other healthful foods^4^	Class I, Level of Evidence A	Grade A, High Level of Certainty	Strong Evidence, Strongly Recommended
To increase consumption of healthful beverages^5^	Class IIb, Level of Evidence B	Grade C, Moderate Level of Certainty	Insufficient Evidence
To reduce BMI	Class IIb, Level of Evidence B	Grade C, Moderate Level of Certainty	Insufficient Evidence
Taxation			
To decrease consumption of SSBs	Class IIa, Level of Evidence B	Grade B, Moderate Level of Certainty	Sufficient Evidence–Recommended
To decrease consumption of unhealthful foods^6^	Class IIb, Level of Evidence B	Grade C, Moderate Level of Certainty	Insufficient Evidence
To reduce BMI	Class IIb, Level of Evidence B	Grade C, Moderate Level of Certainty	Insufficient Evidence

^1^The AHA evidence grading system is: Class I: Conditions for which there is evidence for and/or general agreement that the procedure or treatment is useful and effective; Class II: Conditions for which there is conflicting evidence and/or divergence of opinion about the usefulness/efficacy of a procedure or treatment; Class IIa: Weight of evidence or opinion is in favor of the procedure or treatment; Class IIb: Usefulness/efficacy is less well established by evidence or opinion; Class III: Conditions for which there is evidence and/or general agreement that the procedure or treatment is not useful/effective and in some cases may be harmful. Weight of evidence in support of the recommendation is classified as: Level of Evidence A: Data derived from multiple randomized clinical trials; Level of Evidence B: Data derived from a single randomized trial or nonrandomized studies; Level of Evidence C: Expert opinion or case studies.

^2^The U.S. Preventive Services Task Force is: Grade A: There is high certainty that the net benefit is substantial; Grade B: There is high certainty that the net benefit is moderate or there is moderate certainty that the net benefit is moderate to substantial; Grade C: There is at least moderate certainty that the net benefit is small. Grade D: There is moderate or high certainty that the service has no net benefit or that the harms outweigh the benefits. I Statement: the current evidence is insufficient to assess the balance of benefits and harms of the service. Evidence is lacking, of poor quality, or conflicting, and the balance of benefits and harms cannot be determined. Weight of evidence in support of the recommendation is classified as: High Level of Certainty: the available evidence usually includes consistent results from well-designed, well-conducted studies in representative primary care populations; Moderate Level of Certainty: the available evidence is sufficient to determine the effects of the preventive service on health outcomes, but confidence in the estimate is constrained by such factors as: the number, size, or quality of individual studies, inconsistency of findings across individual studies, limited generalizability of findings to routine primary care practice, lack of coherence in the chain of evidence. Low Level of Certainty: The available evidence is insufficient to assess effects on health outcomes.

^3^CDC Community Guide is: Strong Evidence–Strongly Recommended: good execution, greatest design suitability, at least 2 studies, consistent in direction and size, sufficient effect size, expert opinion not used; Sufficient Evidence–Recommended: good execution, greatest design suitability, 1 study, sufficient effect size, expert opinion not used; Insufficient empirical information supplemented by expert opinion–Recommended based on expert opinion: execution varies, design suitability varies, number of studies varies, and consistency varies, sufficient effect size, expert opinion supports a recommendation; Insufficient Evidence: Available studies do not provide sufficient evidence to assess.

^4^Low fat products, whole grain pizza, dairy products.

^5^ Low fat milk, low calorie beverages.

^6^ Fast foods, energy dense snacks.

## Discussion

Our systematic evaluation of empirical longitudinal evidence on the impact of price changes on diet demonstrates that both subsidies (price decrease) and taxation (price increase) significantly alter dietary consumption of the targeted food items. The majority of evidence was based on interventional studies, and the remainder based on longitudinal evidence on actual price and consumption changes over time, increasing reliance in validity of the results. In addition, compared with cross-sectional modeling studies in which the potential differential effects of the direction of price change (tax vs. subsidy) cannot be assessed, our results identified larger effects on diet of price decreases than price increases: across all items, 12% vs. 6% variation in consumption per 10% price decrease vs. increase, respectively. This investigation is the first, to our knowledge, to determine quantitative effects of price changes on diet based only on interventional and prospective studies.

Several factors could contribute to a greater effect of price subsidies, compared with taxation, on dietary choices. First, interventions promoting healthful behaviors generally have greater effect sizes compared with those targeting cessation of unhealthful behaviors.[54] For example, a meta-analysis on the effectiveness of health communication campaigns for behavior change in the US showed that the effect sizes of the campaigns promoting the commencement of a new positive behavior (e.g., seat belt use, fruits and vegetable consumption) were greater than campaigns promoting the cessation of an existing undesirable behavior (e.g., unsafe sexual behavior, smoking). Almost all interventional studies of price decrease included other components (e.g., promotion/advertising of the price decrease, nutrition education, or changes in availability); although these additional components were not significantly associated with stronger effects, it is possible that these strategies could accentuate the dietary changes achieved by subsidies. It is also possible that methodologic limitations could have led to underestimation of the effects of taxation. Most studies of subsides were interventional and incorporated objective, rigorous assessment of both price changes and dietary changes (e.g., typically based on objective sales data). In contrast, most studies of taxation were observational cohorts, utilizing external databases on average price changes and separately collected information on self-reported dietary intakes. In these latter studies, errors in precision of both the price changes and dietary changes would lead to bias toward the null, causing potential underestimation of the full effects of taxation.

Compared with prior modelling studies,[7, 8] our pooled estimates of price responsiveness were of greater magnitude for fruits and vegetables and of similar magnitude for SSBs. Because these prior studies generally evaluated the cross-sectional relationship between changes in price and consumption, they could not separately assess the potential differential effects of the direction of the price change, as in our investigation. Thus, the findings from prior cross-sectional studies could underestimate the effects of price subsidies (and, similarly, overestimate the effects of taxation). The prospective studies and interventions in our investigation provide evidence on actual dietary changes, but generally did not evaluate complements or substitutes. In contrast, cross-sectional studies can estimate potential complement and substitute effects, but must also estimate the main dietary changes based on modeling. Thus, these two lines of evidence are complementary.

We identified relatively modest differences in price-responsiveness of different food groups beyond the type of price change. Given the scarcity of evidence on the prospective impact of fiscal measures on a range of other dietary factors (e.g., nuts, whole grains, seafood), this finding is important and suggests that food pricing interventions may be an effective policy tool to target diverse food groups.

Our pooled estimates should be considered as the effect of food subsidies or taxation on dietary consumption in relatively stable social settings. Such policies could also be implemented in more dynamic social environments, where multiple factors might be influenced in response to changes in food prices.[55, 56]. Under such circumstances, the effectiveness of food pricing interventions may vary with the relevance and intensity of these external factors and the magnitudes of their interactions with food prices. We also recognize that changes in the price of one food group might influence the consumption of its substitutes and complements (cross-price effect). Most studies included in our investigation did not report sufficient data to evaluate this effect. Our systematic review highlights the need for future interventional and prospective studies evaluating and accounting for multifactorial contexts and cross cross-price effects.

Consistent with their benefits on dietary consumption, we identified a reduction in BMI with price subsidies on healthful foods. While we did not observe a significant effect of price increases on adiposity, the magnitude of the central estimate was similar to that seen for price subsidies; relatively few studies assessed this; and all were observational. These finding suggest potentially limited statistical power to confirm an effect of food taxes on BMI, arguing for additional studies to evaluate this outcome. In long-term studies, dietary changes significantly influence long-term weight gain but with effects that are relatively small among adults not trying to lose weight.[57] Thus, very large and long-term studies may be needed to detect modest but population-relevant effects of price changes on adiposity. Nonetheless, given powerful effects of diet quality on cardiometabolic health, independent of adiposity,[58, 59] improvements in diet are crucial for population health regardless of weight change.

Our investigation has several strengths. We evaluated the empirical evidence from interventional and prospective observational studies. Our systematic search of multiple databases made it less likely that we missed major relevant reports. Full text reviews and data extractions were performed independently and in duplicate, reducing errors or bias and increasing the validity of results. We standardized price changes and dietary changes, allowing quantitative pooling of findings. Our pooled results provide robust estimates of the magnitude of the direct effect of subsidies and taxation on dietary consumption, informing the design and implementation of cost-effective and sustainable fiscal policies. Univariate and multivariate meta-regressions were performed to formally evaluate potential factors that might independently modify the effects. We formally graded the strength of the evidence using established criteria from major organizations.

Potential limitations should be considered. While sales records are more objective than self-reported intakes and are a reasonable proxy, consumption may not always be identical to sales. Evidence on the relationship between taxation and diet mostly came from longitudinal observational studies, in which the possibility of confounding by other social or environmental factors cannot be excluded. Yet, such findings may still provide advantages over cross-sectional observational modeling studies across different population groups. Many studies of subsidies included additional intervention components that might have contributed to their impact. Our evaluation of price change and adiposity was based on few reports, informing the need for additional studies to evaluate this relationship. As with any meta-analysis, evaluation of heterogeneity and publication bias is partly dependent on the total number of studies, and statistical power may have been limited to detect subgroup effects. Most studies were from high-income Western countries, informing the need for additional research in lower-income nations in which fiscal measures might be even more effective.

In conclusion, this systematic review and meta-analysis of interventional and prospective observational studies demonstrates that subsidizing healthful foods significantly increases their consumption; while taxation of unhealthful foods and beverages reduces their intake. Formal appraisal of the strength of evidence identified the highest class of evidence for effectiveness of subsidies to increase fruits and vegetables and other healthful foods; and moderately strong evidence for effects of taxes to reduce SSBs. These findings help to inform the design of fiscal policies, for example including tailored combinations of taxes and subsidies [60] on specific food targets to improve diets and health in populations.

## Supporting information

S1 Fig **Prospective relationship of price decrease (A) and increase (B) with BMI**.
(DOCX)

S2 Fig Begg’s funnel plots for graphical evaluation of potential publication bias. (DOCX)

S1 File PRISMA checklist. (DOCX)

S2 File Study protocol. (DOCX)

S3 File Search query. (DOCX)

S4 File Calculation of the variance of the percent change in the outcome. (DOCX)

S1 Table Quality assessment criteria. (DOCX)

S2 Table Classification of recommendations and level of evidence. (DOCX)

S3 Table Univariate meta-regression models of price change by study characteristics. (DOCX)
