# Supplementary material for: The prospective impact of food pricing on improving dietary consumption: A systematic review and meta-analysis
Source: PLoS One. 2017 Mar 1;12(3):e0172277. doi: 10.1371/journal.pone.0172277 (PMC5332034; doi:10.1371/journal.pone.0172277)
Supplement: S1 Fig — Prospective relationship of price decrease (A) and increase (B) with BMI. (DOCX) [file pone.0172277.s001.docx]

# S1 Fig. Prospective relationship of price decrease (A) and increase (B) with BMI.

**Panel A.**

Study

Change in BMI per 10% price decrease (95%CI)

Design

Weight%

Country

Age Group

**Change in BMI (kg/m^2^) per 10% decrease in price of fruits and vegetables (95%CI)**

**Panel B.**

Study

Change in BMI per 10% price decrease (95%CI)

Design

Weight%

Country

Age Group

**Change in BMI (kg/m^2^) per 10% increase in price of fast food or sugar-sweetened beverages (95%CI)**
