# Supplementary material for: The prospective impact of food pricing on improving dietary consumption: A systematic review and meta-analysis
Source: PLoS One. 2017 Mar 1;12(3):e0172277. doi: 10.1371/journal.pone.0172277 (PMC5332034; doi:10.1371/journal.pone.0172277)
Supplement: S2 Fig — (DOCX) [file pone.0172277.s002.docx]

# S2 Fig. Begg’s funnel plots for graphical evaluation of potential publication bias.

Other Healthful Foods

Fruits & Vegetables

SSBs

Fast Foods
