# Supplementary material for: The prospective impact of food pricing on improving dietary consumption: A systematic review and meta-analysis
Source: PLoS One. 2017 Mar 1;12(3):e0172277. doi: 10.1371/journal.pone.0172277 (PMC5332034; doi:10.1371/journal.pone.0172277)
Supplement: S1 File — (DOCX) [file pone.0172277.s003.docx]

| **Section/topic** | **#** | **Checklist item** | **Reported on page #** | **Text** |
| --- | --- | --- | --- | --- |
| **TITLE** | | | | |
| Title | 1 | Identify the report as a systematic review, meta-analysis, or both. | 1 | The prospective impact of food pricing on improving dietary consumption: a systematic review and meta-analysis |
| **ABSTRACT** | | | | |
| Structured summary | 2 | Provide a structured summary including, as applicable: background; objectives; data sources; study eligibility criteria, participants, and interventions; study appraisal and synthesis methods; results; limitations; conclusions and implications of key findings; systematic review registration number. | 2,3 | Please see the ABSTRACT |
| **INTRODUCTION** | | | | |
| Rationale | 3 | Describe the rationale for the review in the context of what is already known. | 4 | Several reviews suggest that price changes may prospectively improve diet and obesity; yet, this evidence has been summarized only qualitatively, without quantitative assessment of effectiveness or key potential sources of heterogeneity. |
| Objectives | 4 | Provide an explicit statement of questions being addressed with reference to participants, interventions, comparisons, outcomes, and study design (PICOS). | 4 | To systematically investigate and quantify the prospective effect of change in food price on dietary consumption, and how key additional interventions might modify these effects. |
| **METHODS** | | | | |
| Protocol and registration | 5 | Indicate if a review protocol exists, if and where it can be accessed (e.g., Web address), and, if available, provide registration information including registration number. | 5 | The study objective, search strategy, and selection criteria were specified in advance in the Study Protocol (S2 File). |
| Eligibility criteria | 6 | Specify study characteristics (e.g., PICOS, length of follow-up) and report characteristics (e.g., years considered, language, publication status) used as criteria for eligibility, giving rationale. | 6 | Please see Study Selection |
| Information sources | 7 | Describe all information sources (e.g., databases with dates of coverage, contact with study authors to identify additional studies) in the search and date last searched. | 5, 6 | We searched multiple online databases including PubMed, Econlit, Embase, Ovid, Cochrane Library, Web of Science, and CINAHL.  For each of the articles included in the final analysis as well as the relevant reviews identified through search of databases, we hand-searched the reference list and the first 20 “related articles” in PubMed.  The date of search for each database is provided in S3 File. |
| Search | 8 | Present full electronic search strategy for at least one database, including any limits used, such that it could be repeated. | 5, 6 | Search terms were compiled in 3 categories: setting queries (e.g., national, state, city, workplace, schools, supermarket, restaurant, fast food, and cafeteria), intervention queries (e.g., tax, subsidy, incentive, and price) and outcome queries (e.g., food, beverage, fruit, vegetable, soda, meat, dairy, overweight, obesity, and adiposity).  The complete list of the search terms are provided in the S3 File. |
| Study selection | 9 | State the process for selecting studies (i.e., screening, eligibility, included in systematic review, and, if applicable, included in the meta-analysis). | 6 | Please see Study Selection |
| Data collection process | 10 | Describe method of data extraction from reports (e.g., piloted forms, independently, in duplicate) and any processes for obtaining and confirming data from investigators. | 6, 7 | Using a standardized electronic format, 2 investigators extracted data independently and in duplicate.  Differences in data extraction between investigators were infrequent and were resolved by consensus. |
| Data items | 11 | List and define all variables for which data were sought (e.g., PICOS, funding sources) and any assumptions and simplifications made. | 6 | Data were extracted on first author name, publication year, study location, design, population (age, sex, race, sample size), duration of follow-up, price data , outcome data (definition, ascertainment methods, change), and (for observational studies) covariates. |
| Risk of bias in individual studies | 12 | Describe methods used for assessing risk of bias of individual studies (including specification of whether this was done at the study or outcome level), and how this information is to be used in any data synthesis. | 6, 7 | Two investigators independently assessed the quality of studies based on 5 criteria: study design, assessment of exposure, assessment of outcome, control for confounding, and evidence of selection bias (S1 Table). For each criterion, each study received a score of 1 or 0 (1 being better), and an overall quality score was calculated as the sum of individual scores.  Differences in quality assessment between investigators were infrequent and were resolved by consensus.  Meta-regression (metareg command in Stata) was used to explore potential heterogeneity by quality score (continuous). |
| Summary measures | 13 | State the principal summary measures (e.g., risk ratio, difference in means). | 7 | The primary outcome was the percent change in consumption of foods/beverages due to the percent change in their price. For pooling, each study-specific effect was standardized to a 10% price change, assuming a linear dose-response relationship. |
| Synthesis of results | 14 | Describe the methods of handling data and combining results of studies, if done, including measures of consistency (e.g., I^2^) for each meta-analysis. | 7 | Absolute consumption or absolute price changes were not combined due to heterogeneity in currencies, base prices, and base consumptions. Studies only reporting absolute price changes, without required information to calculate percentage change, were only included qualitative assessment of the evidence. The variance of percent change in consumption was calculated based on the variance of the outcome at baseline and end-follow up, assuming a correlation between these measures of 0.5. Study-specific effect sizes were pooled using inverse-variance-weighted random-effect models (metan command in Stata). Cochran's Q and the I2 were used to assess the between-study heterogeneity; with I2 values of 25%, 50%, and 75% representing low, moderate, and high heterogeneity. |
| Risk of bias across studies | 15 | Specify any assessment of risk of bias that may affect the cumulative evidence (e.g., publication bias, selective reporting within studies). | 7, 8 | Publication bias was assessed by visual inspection of funnel plots, Egger's test, and Begg's test. |
| Additional analyses | 16 | Describe methods of additional analyses (e.g., sensitivity or subgroup analyses, meta-regression), if done, indicating which were pre-specified. | 7 | Meta-regression (metareg command in Stata) was used to explore potential sources of heterogeneity including population (adults, children), design (randomized, nonrandomized, observational), country (US, non–US), direction of price change (increase, decrease), additional components (price change only, multicomponent), type of additional components (changes in availability, promotion/advertising of price change, labeling, nutrition education), follow-up duration (months), and quality score (continuous). |
| **RESULTS** | | | | |
| Study selection | 17 | Give numbers of studies screened, assessed for eligibility, and included in the review, with reasons for exclusions at each stage, ideally with a flow diagram. |  | Please see Fig 1 |
| Study characteristics | 18 | For each study, present characteristics for which data were extracted (e.g., study size, PICOS, follow-up period) and provide the citations. |  | Please see Table 1 and Table 2 |
| Risk of bias within studies | 19 | Present data on risk of bias of each study and, if available, any outcome level assessment (see item 12). |  | Please see Table 1 |
| Results of individual studies | 20 | For all outcomes considered (benefits or harms), present, for each study: (a) simple summary data for each intervention group (b) effect estimates and confidence intervals, ideally with a forest plot. |  | Please see Fig 2 and S1 Fig |
| Synthesis of results | 21 | Present results of each meta-analysis done, including confidence intervals and measures of consistency. |  | Please see Fig 2 and S1 Fig |
| Risk of bias across studies | 22 | Present results of any assessment of risk of bias across studies (see Item 15). | 18 | Visual inspection of funnel plots provided mixed evidence for publication bias (S2 Fig). However, Begg’s or Eggers test did not identify statistical evidence for publication bias, although numbers of studies in some of these analyses were limited. |
| Additional analysis | 23 | Give results of additional analyses, if done (e.g., sensitivity or subgroup analyses, meta-regression [see Item 16]). | 17, 18 | In univariate meta-regression, findings were not significantly different according to differences in study design (randomized intervention, nonrandomized intervention, prospective cohort), location (US, other), setting (cafeteria, community, supermarket, vending machine) duration (months), population (adults, children, both), number of additional intervention components (none, 1-2) type of additional intervention component (none, change in food availability, labeling, nutrition education, food promotion) (P>0.05 each; S3 Table). Statistically significant larger effects were identified in studies with price decreases (subsidies) vs. increases (taxes) (P-heterogeneity=0.044); and with lower (2-3) vs. higher (4-5) study quality score (P-heterogeneity=0.034). In multivariate meta-regression including direction of price change and study quality score simultaneously, neither was statistically significant due to collinearity. |
| **DISCUSSION** | | | | |
| Summary of evidence | 24 | Summarize the main findings including the strength of evidence for each main outcome; consider their relevance to key groups (e.g., healthcare providers, users, and policy makers). | 22 | Our systematic evaluation of empirical longitudinal evidence on the impact of price changes on diet demonstrates that both subsidies (price decrease) and taxation (price increase) significantly alter dietary consumption of the targeted food items. The majority of evidence was based on interventional studies, and the remainder based on longitudinal evidence on actual price and consumption changes over time, increasing reliance in validity of the results. In addition, compared with cross-sectional modeling studies in which the potential differential effects of the direction of price change (tax vs. subsidy) cannot be assessed, our results identified larger effects on diet of price decreases than price increases: across all items, 12% vs. 6% variation in consumption per 10% price decrease vs. increase, respectively. This investigation is the first, to our knowledge, to determine quantitative effects of price changes on diet based only on interventional and prospective studies |
| Limitations | 25 | Discuss limitations at study and outcome level (e.g., risk of bias), and at review-level (e.g., incomplete retrieval of identified research, reporting bias). | 25,26 | Potential limitations should be considered. While sales records are more objective than self-reported intakes and are a reasonable proxy, consumption may not always be identical to sales. Evidence on the relationship between taxation and diet mostly came from longitudinal observational studies, in which the possibility of confounding by other social or environmental factors cannot be excluded. Yet, such findings may still provide advantages over cross-sectional observational modeling studies across different population groups. Many studies of subsidies included additional intervention components that might have contributed to their impact. Our evaluation of price change and adiposity was based on few reports, informing the need for additional studies to evaluate this relationship. As with any meta-analysis, evaluation of heterogeneity and publication bias is partly dependent on the total number of studies, and statistical power may have been limited to detect subgroup effects. Most studies were from high-income Western countries, informing the need for additional research in lower-income nations in which fiscal measures might be even more effective. |
| Conclusions | 26 | Provide a general interpretation of the results in the context of other evidence, and implications for future research. | 26 | In conclusion, this systematic review and meta-analysis of interventional and prospective observational studies demonstrates that subsidizing healthful foods significantly increases their consumption; while taxation of unhealthful foods and beverages reduces their intake. Formal appraisal of the strength of evidence identified the highest class of evidence for effectiveness of subsidies to increase fruits and vegetables and other healthful foods; and moderately strong evidence for effects of taxes to reduce SSBs. These findings help to inform the design of fiscal policies, for example including tailored combinations of taxes and subsidies on specific food targets to improve diets and health in populations. |
| **FUNDING** | | | | |
| Funding | 27 | Describe sources of funding for the systematic review and other support (e.g., supply of data); role of funders for the systematic review. |  | AA was supported by T32 HL098048 from the National Heart, Lung, and Blood Institute. DM was supported by R01 HL115189 from the National Heart, Lung, and Blood Institute and a Research Award from The New York Academy of Sciences' Sacker Institute for Nutrition Science. JP was partly supported by a Bunge Fellowship in Global Nutrition. DM reports ad hoc travel reimbursement or honoraria from Bunge, Pollock Institute, Quaker Oats, and Life Sciences Research Organization; ad hoc consulting fees from McKinsey Health Systems Institute, Foodminds, Nutrition Impact, Amarin, Omthera, and Winston and Strawn LLP; membership, Unilever North America Scientific Advisory Board; royalties from UpToDate; and research grants from GlaxoSmithKline, Sigma Tau, Pronova, the Gates Foundation, the Sackler Institute of Nutrition, and the National Institutes of Health. The funders had no role in study design, data collection and analysis, decision to publish, or preparation of the manuscript. |

*From:*  Moher D, Liberati A, Tetzlaff J, Altman DG, The PRISMA Group (2009). Preferred Reporting Items for Systematic Reviews and Meta-Analyses: The PRISMA Statement. PLoS Med 6(6): e1000097. doi:10.1371/journal.pmed1000097

For more information, visit: **www.prisma-statement.org**.
