# Supplementary material for: The prospective impact of food pricing on improving dietary consumption: A systematic review and meta-analysis
Source: PLoS One. 2017 Mar 1;12(3):e0172277. doi: 10.1371/journal.pone.0172277 (PMC5332034; doi:10.1371/journal.pone.0172277)
Supplement: S2 File — (DOCX) [file pone.0172277.s004.docx]

# Study Protocol

**Objective**

To systematically review and quantify the prospective effect of food pricing on diet

**Methods**

The recommendations of the Meta-analysis of Observational Studies in Epidemiology (MOOSE) and of Preferred Reporting Items for Systematic reviews and Meta-Analyses (PRISMA) guidelines will be followed for observational studies and randomized control trials (RCTs), respectively, during all stages of the design, implementation, and reporting of this meta-analysis.

**Definition of Exposure and Outcomes:**

*Exposure/Intervention:*

1. Change in food/beverage price due to taxation, subsides, or other factors

*Outcome:*

1. Change in Diet: Change in intake of foods and beverages (e.g., fruits, vegetables, whole grains, nuts, dietary fiber, milk, sugar-sweetened beverages, processed meats, salt, and trans-fat). Sales/purchases data will be considered as proxy for change in food consumption.
2. Other outcomes include change in body weight and body mass index.

**Inclusion Criteria**

1. *Design:* All experimental (RCTs and quasi-experimental) and prospective observational studies that assessed the association between food price and diet or adiposity.
2. *Population:* Any (adults and children)
3. *Setting:* Any (e.g., nationwide, statewide, or at the level of city, community, workplace, or school).
4. *Exposure:* Change in food/beverage price due to taxation, subsides, or other factors
5. *Outcome:* Change in intake of foods/beverages, sales/purchases of foods/beverages, and adiposity (e.g., body weight, and body mass index).
6. *Effect Measure:* Studies have to provide an estimate of the change in dietary behavior or adiposity and a measure of uncertainty for the reported change.

**Exclusion Criteria**

1. *Type of Articles*: Commentaries, general reviews, duplicate publications from the same study. But commentaries and reviews will be included in the initial screening as a source of references. Duplicate publications from the same study will also be included in the initial screening for further assessment of the full-text. In such cases, the decision will be made after assessment of the full-text of the articles and based on the quality of each study.
2. *Design:* Ecological studies (across the nations e.g., food price in Japan vs. US), lab experiments (hypothetical situations), or simulation studies (modelling).
3. *Exposure:* Exposure data collection before 1990.
4. *Outcome:* Physiologic measures other than adiposity.
5. *Effect Measure*: Observational studies that included only crude estimates.
