# Supplementary material for: The prospective impact of food pricing on improving dietary consumption: A systematic review and meta-analysis
Source: PLoS One. 2017 Mar 1;12(3):e0172277. doi: 10.1371/journal.pone.0172277 (PMC5332034; doi:10.1371/journal.pone.0172277)
Supplement: S3 File — (DOCX) [file pone.0172277.s005.docx]

# Search Query for PubMed/Medline

Setting Query

("National"[tiab] OR "Nationwide"[tiab] OR "state"[tiab] OR "statewide"[tiab] OR "city"[tiab] OR "Workplace"[MeSH Major Topic] OR "Workplace"[tiab] OR "Schools"[MeSH Terms] OR "School"[tiab] OR "School$"[tiab] OR “Supermarket$”[tiab] OR “restaurant$”[tiab] OR “fast food”[tiab] OR “store$” [tiab] OR “cafe” [tiab] OR “cafeteria”[tiab])

Intervention/Exposure Query

(“taxes”[MeSH Terms] OR "tax"[tiab] OR "taxation"[tiab] OR "subsidy"[tiab] OR "subsidies"[tiab] OR "incentive"[tiab] OR "price"[tiab] OR "pricing"[tiab] OR "voucher"[tiab] OR "coupon"[tiab] OR "rebate"[tiab] OR “elasticity”[tiab] OR “elasticities”[tiab])

Outcome Query

(“Food and Beverages” [MeSH Terms] OR "fruit"[MeSH Terms] OR "fruit"[tiab] OR "vegetables"[MeSH Terms] OR "vegetables"[tiab] OR "fat"[tiab] OR “Sugar-sweetened beverage”[tiab] OR “soda”[tiab] OR “meat”[tiab] OR “dairy”[tiab] OR “candy”[tiab] OR "obesity"[MeSH Terms] OR "obesity"[tiab] OR “BMI”[tiab] OR "body weight"[MeSH Terms] OR "sodium, dietary"[MeSH Terms] OR "sodium"[tiab] OR "Body Mass Index"[MeSH Major Topic] OR "Adiposity"[MeSH Major Topic] OR "Adiposity"[tiab] OR “food consumption” [tiab] OR “Overweight” [MeSH] OR “Overweight” [tiab] OR “calorie”[tiab] OR “calorie$”[tiab])

All Queries

("National"[tiab] OR "Nationwide"[tiab] OR "state"[tiab] OR "statewide"[tiab] OR "city"[tiab] OR "Workplace"[MeSH Major Topic] OR "Workplace"[tiab] OR "Schools"[MeSH Terms] OR "School"[tiab] OR "School$"[tiab] OR “Supermarket$”[tiab] OR “restaurant$”[tiab] OR “fast food”[tiab] OR “store$” [tiab] OR “cafe” [tiab] OR “cafeteria”[tiab])

AND

(“taxes”[MeSH Terms] OR "tax"[tiab] OR "taxation"[tiab] OR "subsidy"[tiab] OR "subsidies"[tiab] OR "incentive"[tiab] OR "price"[tiab] OR "pricing"[tiab] OR "voucher"[tiab] OR "coupon"[tiab] OR "rebate"[tiab] OR “elasticity”[tiab] OR “elasticities”[tiab])

AND

(“Food and Beverages” [MeSH Terms] OR "fruit"[MeSH Terms] OR "fruit"[tiab] OR "vegetables"[MeSH Terms] OR "vegetables"[tiab] OR "fat"[tiab] OR “Sugar-sweetened beverage”[tiab] OR “soda”[tiab] OR “meat”[tiab] OR “dairy”[tiab] OR “candy”[tiab] OR "obesity"[MeSH Terms] OR "obesity"[tiab] OR “BMI”[tiab] OR "body weight"[MeSH Terms] OR "sodium, dietary"[MeSH Terms] OR "sodium"[tiab] OR "Body Mass Index"[MeSH Major Topic] OR "Adiposity"[MeSH Major Topic] OR "Adiposity"[tiab] OR “food consumption” [tiab] OR “Overweight” [MeSH] OR “Overweight” [tiab] OR “calorie”[tiab] OR “calorie$”[tiab])

Limits

Age Any

Setting Any country

Year Range 1990

Language Any

Abstracts identified: 720 Hits

Date: 6/5/2014

# Search Query for EconLit

Setting Query

(ti(National OR Nationwide OR state OR statewide OR city OR Workplace OR Schools OR Supermarket OR restaurant OR fast food OR store OR cafe OR cafeteria) OR ab(National OR Nationwide OR state OR statewide OR city OR Workplace OR Schools OR Supermarket OR restaurant OR fast food OR store OR cafe OR cafeteria))

Intervention/Exposure Query

(ti(tax OR taxation OR subsidy OR subsidies OR incentive OR price OR pricing OR voucher OR coupon OR rebate OR elasticity) OR ab(tax OR taxation OR subsidy OR subsidies OR incentive OR price OR pricing OR voucher OR coupon OR rebate OR elasticity))

Outcome Query

(ti (Foods OR Beverages OR fruits OR vegetables OR fat OR soda OR meat OR dairy OR candy OR obesity OR Adiposity OR Overweight OR Calorie) OR ab(Foods OR Beverages OR fruits OR vegetables OR fat OR soda OR meat OR dairy OR candy OR obesity OR Adiposity OR Overweight OR Calorie))

All Queries

(ti(National OR Nationwide OR state OR statewide OR city OR Workplace OR Schools OR Supermarket OR restaurant OR fast food OR store OR cafe OR cafeteria) OR ab(National OR Nationwide OR state OR statewide OR city OR Workplace OR Schools OR Supermarket OR restaurant OR fast food OR store OR cafe OR cafeteria)) AND (ti(tax OR taxation OR subsidy OR subsidies OR incentive OR price OR pricing OR voucher OR coupon OR rebate OR elasticity) OR ab(tax OR taxation OR subsidy OR subsidies OR incentive OR price OR pricing OR voucher OR coupon OR rebate OR elasticity)) AND (ti (Foods OR Beverages OR fruits OR vegetables OR fat OR soda OR meat OR dairy OR candy OR obesity OR Adiposity OR Overweight OR Calorie) OR ab(Foods OR Beverages OR fruits OR vegetables OR fat OR soda OR meat OR dairy OR candy OR obesity OR Adiposity OR Overweight OR Calorie))

Limits

Age Any

Setting Any country

Year Range 1990

Language Any

Abstracts identified: 1504

Date: 6/5/2014

**Search Query for OVID**

Setting Query

(National OR Nationwide OR state OR statewide OR city OR Workplace OR Schools OR Supermarket OR restaurant OR fast food OR store OR cafe OR cafeteria)

Intervention/Exposure Query

(tax OR taxation OR subsidy OR subsidies OR incentive OR price OR pricing OR voucher OR coupon OR rebate OR elasticity)

Outcome Query

(Foods OR Beverages OR fruits OR vegetables OR fat OR soda OR meat OR dairy OR candy OR obesity OR Adiposity OR Overweight OR Calorie)

All Queries

((National or Nationwide or state or statewide or city or Workplace or Schools or Supermarket or restaurant or fast food or store or cafe or cafeteria) and (tax or taxation or subsidy or subsidies or incentive or price or pricing or voucher or coupon or rebate or elasticity) and (Foods or Beverages or fruits or vegetables or fat or soda or meat or dairy or candy or obesity or Adiposity or Overweight or Calorie)).ab.

Limits

Age Any

Setting Any country

Year Range 1990

Language Any

Abstracts identified: 4

Date: 6/5/2014

# Search Query for Embase

Setting Query

(National OR Nationwide OR state OR statewide OR city OR Workplace OR Schools OR Supermarket OR restaurant OR fast food OR store OR cafe OR cafeteria)

Intervention/Exposure Query

(tax OR taxation OR subsidy OR subsidies OR incentive OR price OR pricing OR voucher OR coupon OR rebate OR elasticity)

Outcome Query

(Foods OR Beverages OR fruits OR vegetables OR fat OR soda OR meat OR dairy OR candy OR obesity OR Adiposity OR Overweight OR Calorie)

All Queries

(National OR Nationwide OR state OR statewide OR city OR Workplace OR Schools OR Supermarket OR restaurant OR fast food OR store OR cafe OR cafeteria) AND (tax OR taxation OR subsidy OR subsidies OR incentive OR price OR pricing OR voucher OR coupon OR rebate OR elasticity) AND (Foods OR Beverages OR fruits OR vegetables OR fat OR soda OR meat OR dairy OR candy OR obesity OR Adiposity OR Overweight OR Calorie)

Limits

Age Any

Setting Any country

Year Range 1990

Language Any

Abstracts identified: 1277

Date: 6/5/2014

# Search Query for CINHAL

Setting Query

(National OR Nationwide OR state OR statewide OR city OR Workplace OR Schools OR Supermarket OR restaurant OR fast food OR store OR cafe OR cafeteria)

Intervention/Exposure Query

(tax OR taxation OR subsidy OR subsidies OR incentive OR price OR pricing OR voucher OR coupon OR rebate OR elasticity)

Outcome Query

(Foods OR Beverages OR fruits OR vegetables OR fat OR soda OR meat OR dairy OR candy OR obesity OR Adiposity OR Overweight OR Calorie)

All Queries

AB ((National OR Nationwide OR state OR statewide OR city OR Workplace OR Schools OR Supermarket OR restaurant OR fast food OR store OR cafe OR cafeteria) ) AND AB ( (tax OR taxation OR subsidy OR subsidies OR incentive OR price OR pricing OR voucher OR coupon OR rebate OR elasticity) ) AND AB ( (Foods OR Beverages OR fruits OR vegetables OR fat OR soda OR meat OR dairy OR candy OR obesity OR Adiposity OR Overweight OR Calorie))

Limits

Age Any

Setting Any country

Year Range 1990

Language Any

Abstracts identified: 113

Date: 6/5/2014

# Search Query for Cochrane Library

Setting Query

(National OR Nationwide OR state OR statewide OR city OR Workplace OR Schools OR Supermarket OR restaurant OR fast food OR store OR cafe OR cafeteria)

Intervention/Exposure Query

(tax OR taxation OR subsidy OR subsidies OR incentive OR price OR pricing OR voucher OR coupon OR rebate OR elasticity)

Outcome Query

(Foods OR Beverages OR fruits OR vegetables OR fat OR soda OR meat OR dairy OR candy OR obesity OR Adiposity OR Overweight OR Calorie)

All Queries

((National OR Nationwide OR state OR statewide OR city OR Workplace OR Schools OR Supermarket OR restaurant OR fast food OR store OR cafe OR cafeteria)) AND ((tax OR taxation OR subsidy OR subsidies OR incentive OR price OR pricing OR voucher OR coupon OR rebate OR elasticity)) AND ((Foods OR Beverages OR fruits OR vegetables OR fat OR soda OR meat OR dairy OR candy OR obesity OR Adiposity OR Overweight OR Calorie))

Limits

Age Any

Setting Any country

Year Range 1990

Language Any

Abstracts identified: 0

Date: 6/5/2014

# Search Query for Web of Science

Setting Query

(TS=(National OR Nationwide OR state OR statewide OR city OR Workplace OR Schools OR Supermarket OR restaurant OR fast food OR store OR cafe OR cafeteria)

Intervention/Exposure Query

(TS=(tax OR taxation OR subsidy OR subsidies OR incentive OR price OR pricing OR voucher OR coupon OR rebate OR elasticity)

Outcome Query

(TS=(Foods OR Beverages OR fruits OR vegetables OR fat OR soda OR meat OR dairy OR candy OR obesity OR Adiposity OR Overweight OR Calorie)

All Queries

TI=(National OR Nationwide OR state OR statewide OR city OR Workplace OR Schools OR Supermarket OR restaurant OR fast food OR store OR cafe OR cafeteria)) AND (TI=(tax OR taxation OR subsidy OR subsidies OR incentive OR price OR pricing OR voucher OR coupon OR rebate OR elasticity)) AND (TI=(Foods OR Beverages OR fruits OR vegetables OR fat OR soda OR meat OR dairy OR candy OR obesity OR Adiposity OR Overweight OR Calorie))

Limits

Age Any

Setting Any country

Year Range 1990

Language Any

Abstracts identified: 161

Date: 6/5/2014
