# Supplementary material for: The prospective impact of food pricing on improving dietary consumption: A systematic review and meta-analysis
Source: PLoS One. 2017 Mar 1;12(3):e0172277. doi: 10.1371/journal.pone.0172277 (PMC5332034; doi:10.1371/journal.pone.0172277)
Supplement: S4 File — (DOCX) [file pone.0172277.s006.docx]

# Calculation of the Variance of the Percent Change in the Outcome

We used the following formula to calculated variance of the percent change in the outcome:

$$Var\left( \frac{X_{2}-X_{1}}{X_{1}} \right)=Var \left( \frac{X_{2}}{X_{1}}-1 \right)=Var\left( \frac{X_{2}}{X_{1}} \right)$$

$$Var\left( \frac{X_{2}}{X_{1}} \right)\sim\frac{1}{{X_{1}}^{2}}Var\left[ X_{2} \right]+\frac{{X_{2}}^{2}}{{X_{1}}^{4}}Var\left[ X_{1} \right]-2\frac{X_{2}}{{X_{1}}^{3}}Cov\left[ X_{1},X_{2} \right]$$

X_1_=mean intake of dietary factor at baseline

X_2_= mean intake of dietary factor at the end of follow up

The correlation coefficient (ρ) of 0.5 was used to calculate the covariance between mean intake at baseline and at the end of follow up using the following formula:

$$Cov\left[ X_{1},X_{2} \right]=\rho_{X_{1},X_{2}}\times\sigma_{X_{1}}\times\sigma_{X_{2}}$$

In different sensitivity analyses, we examined the effect of the change in correlation coefficient (range: 0.1-0.9) on the weights of the studies and the pooled estimates and did not find any significant effect.
