# Supplementary material for: The prospective impact of food pricing on improving dietary consumption: A systematic review and meta-analysis
Source: PLoS One. 2017 Mar 1;12(3):e0172277. doi: 10.1371/journal.pone.0172277 (PMC5332034; doi:10.1371/journal.pone.0172277)
Supplement: S1 Table — (DOCX) [file pone.0172277.s007.docx]

| **S1 Table**. Quality assessment criteria | | | |
| --- | --- | --- | --- |
| **Criterion** | **Range** | **Description** | |
| Design | 0-1 | 1 | if interventional (randomized or non-randomized) trial |
|  |  | 0 | if observational study |
| Assessment of intervention/exposure | 0-1 | 1 | if single component intervention; multicomponent intervention separately reporting the effect size of each component; or observational study accurately assessing exposure level |
|  |  | 0 | if multicomponent intervention not separately reporting the effect size of each component; or observational study using non-optimal methods for exposure assessment |
| Assessment of outcome | 0-1 | 1 | if individual total intake assessed by a valid method (e.g., FFQ, 24-hr recall, food diary) or individual level data used for obesity measures |
|  |  | 0 | if only individual intake assessed at a given setting (e.g., using sales record) or state-level data used for obesity measures |
| Control for confounding | 0-1 | 1 | if RCT or sufficient control for major individual level confounders |
|  |  | 0 | if insufficient control for major confounders |
| Evidence of selection bias | 0-1 | 1 | if absence of evidence for selection bias (e.g., differential loss to follow up) |
|  |  | 0 | if substantial presence of evidence for selection bias |
