# Supplementary material for: The prospective impact of food pricing on improving dietary consumption: A systematic review and meta-analysis
Source: PLoS One. 2017 Mar 1;12(3):e0172277. doi: 10.1371/journal.pone.0172277 (PMC5332034; doi:10.1371/journal.pone.0172277)
Supplement: S2 Table — (DOCX) [file pone.0172277.s008.docx]

|  | |  |
| --- | --- | --- |
| **S2 Table**. Classification of recommendations and level of evidence | |  |
| Recommendation Classification | Weight of the Evidence | |
| **American Heart Association^1^** |  | |
| **Class I:** There is evidence for and general agreement that the intervention is beneficial, useful, and effective. The intervention should be performed. | **Level of Evidence A:** Data derived from multiple randomized clinical trials or meta-analyses *or, given the nature of the population interventions, from well-designed quasi-experimental studies combine with supportive evidence from several other types of studies.* | |
| **Class II:** There is conflicting evidence and/or a divergence of opinion about the usefulness/efficacy of the intervention. | **Level of Evidence B:** Data derived from a single randomized trial or nonrandomized studies. | |
| **Class IIa:** Weight of evidence/opinion is in favor of usefulness/efficacy. It is reasonable to perform the intervention. | **Level of Evidence C:** Only consensus of opinion of experts, case studies, or standard of care. | |
| **Class IIb:** Usefulness/efficacy is less well established by evidence/opinion. The intervention may be considered. |  | |
| **Class III:** There is evidence and/or general agreement that the intervention is not useful/effective and in some cases may be harmful. |  | |
|  |  | |
| **U.S. Preventive Services Task Force^2^** |  | |
| **Grade A:** The USPSTF recommends the service. There is high certainty that the net benefit is substantial. Offer or provide this service. | **High Level of Certainty:** The available evidence usually includes consistent results from well-designed, well-conducted studies in representative primary care populations. These studies assess the effects of the preventive service on health outcomes. This conclusion is therefore unlikely to be strongly affected by the results of future studies. | |
| **Grade B:** The USPSTF recommends the service. There is high certainty that the net benefit is moderate or there is moderate certainty that the net benefit is moderate to substantial. Offer or provide this service. | **Moderate Level of Certainty:** The available evidence is sufficient to determine the effects of the preventive service on health outcomes, but confidence in the estimate is constrained by such factors as: the number, size, or quality of individual studies; inconsistency of findings across individual studies; limited generalizability of findings to routine primary care practice; lack of coherence in the chain of evidence. As more information becomes available, the magnitude or direction of the observed effect could change, and this change may be large enough to alter the conclusion. | |
| **Grade C:** The USPSTF recommends selectively offering or providing this service to individual patients based on professional judgment and patient preferences. There is at least moderate certainty that the net benefit is small. Offer or provide this service for selected patients depending on individual circumstances. | **Low Level of Certainty:** The available evidence is insufficient to assess effects on health outcomes. Evidence is insufficient because of: the limited number or size of studies; important flaws in study design or methods; inconsistency of findings across individual studies; gaps in the chain of evidence; findings not generalizable to routine primary care practice, lack of information on important health outcomes. More information may allow estimation of effects on health outcomes. | |
| **Grade D:** The USPSTF recommends against the service. There is moderate or high certainty that the service has no net benefit or that the harms outweigh the benefits. Discourage the use of this service. |  | |
| **I Statement:** The USPSTF concludes that the current evidence is insufficient to assess the balance of benefits and harms of the service. Evidence is lacking, of poor quality, or conflicting, and the balance of benefits and harms cannot be determined. Read the clinical considerations section of USPSTF Recommendation Statement. If the service is offered, patients should understand the uncertainty about the balance of benefits and harms. |  | |
|  |  | |
| **CDC Community Guide^3,4^** |  | |
| **Recommended:** The systematic review of available studies provides strong or sufficient evidence that the intervention is effective. The categories of “strong” and “sufficient” evidence reflect the Task Force’s degree of confidence that an intervention has beneficial effects. They do not directly relate to the expected magnitude of benefits. The categorization is based on several factors such as study design, number of studies, and consistency of the effect across studies. | **Strong Evidence – Strongly Recommended**  1. Good execution, greatest design suitability, at least 2 studies, consistent in direction and size, sufficient effect size, expert opinion not used  2. Good execution, greatest or moderate design suitability, at least 5 studies, consistent in direction and size, sufficient effect size, expert opinion not used  3. Good or fair execution, greatest design suitability, at least 5 studies, consistent in direction and size, sufficient effect size, expert opinion not used  4. Meets design, execution, number and consistency criteria for sufficient evidence, large effect size, expert opinion not used | |
| **Recommended Against:** The systematic review of available studies provides strong or sufficient evidence that the intervention is harmful or not effective. | **Sufficient Evidence – Recommended**  1. Good execution, greatest design suitability, 1 study, sufficient effect size, expert opinion not used  2. Good or fair execution, greatest or moderate design suitability, at least 3 studies, consistent direction and size, sufficient effect size, expert opinion not used  3. Good or fair execution, greatest, moderate, or least design suitability, at least 5 studies, consistent direction and size, sufficient effect size, expert opinion not used | |
| **Insufficient Evidence:** The available studies do not provide sufficient evidence to determine if the intervention is, or is not, effective. This does not mean that the intervention does not work. It means that additional research is needed to determine whether or not the intervention is effective. | **Insufficient empirical information supplemented by expert opinion – Recommended based on expert opinion**  1. Execution varies, design suitability varies, number of studies varies, and consistency varies, sufficient effect size, expert opinion supports a recommendation | |
|  | **Insufficient Evidence – Available studies do not provide sufficient evidence to assess**  5 categories that are not mutually exclusive – one or more of these will occur when a body of evidence fails to meet the criteria for strong or sufficient evidence:  1. Insufficient Designs or Execution 2. Too Few Studies 3. Inconsistent Direction or Size 4. Small effect size 5. Expert opinion not used | |
|  | **Sufficient or strong evidence of ineffectiveness or harm – Recommendation is discouraged** | |

1. Mozaffarian D, Afshin A, Benowitz NL, et al. Population approaches to improve diet, physical activity, and smoking habits: a scientific statement from the American Heart Association. Circulation 2012;126(12):1514-63.

2. U.S. Preventive Services Task Force. U.S. Preventive Services Task Force Grade Definitions. Secondary U.S. Preventive Services Task Force Grade Definitions 2013. http://www.uspreventiveservicestaskforce.org/uspstf/grades.htm.

3. Briss PA, Zaza S, Pappaioanou M, et al. Developing an evidence-based Guide to Community Preventive Services--methods. The Task Force on Community Preventive Services. Am J Prev Med 2000;18(1 Suppl):35-43.

4. The Guide to Community Preventive Services. Secondary The Guide to Community Preventive Services. http://www.thecommunityguide.org/about/methods.html.
