# Supplementary material for: The prospective impact of food pricing on improving dietary consumption: A systematic review and meta-analysis
Source: PLoS One. 2017 Mar 1;12(3):e0172277. doi: 10.1371/journal.pone.0172277 (PMC5332034; doi:10.1371/journal.pone.0172277)
Supplement: S3 Table — (DOCX) [file pone.0172277.s009.docx]

| **S3 Table**. Univariate meta-regression models of price change by study characteristics | | | | |
| --- | --- | --- | --- | --- |
|  |  |  |  |  |
| **Study characteristic** |  | **N** | **Elasticity (95%CI)^1^** | **p-value** |
|  |  |  |  |  |
| **Overall** |  | 37 | -0.95 (-1.1, -0.78) | 0.000 |
|  |  |  |  |  |
| **Design** | Non-random intervention | 21 | (reference) | - |
|  | Randomized intervention | 10 | 0.31 (-0.56, 1.18) | 0.476 |
|  | Prospective cohort | 6 | 1.02 (-0.02, 2.05) | 0.055 |
|  |  |  |  |  |
| **Location** | United States | 29 | (reference) |  |
|  | Other | 8 | 0.36 (-0.56, 1.28) | 0.434 |
|  |  |  |  |  |
| **Setting ^2^** | Cafeteria | 14 |  |  |
|  | Community | 6 | 0.78 (-0.07, 1.62) | 0.069 |
|  | Supermarket | 8 | 0.09 (-0.7, 0.88) | 0.816 |
|  | Vending machine | 7 | 0.03 (-0.78, 0.84) | 0.947 |
|  |  |  |  |  |
| **Duration** | Per month | 19 | 0 (0, 0.01) | 0.192 |
|  |  |  |  |  |
| **Population** | Adults | 27 | (reference) | - |
|  | Children | 3 | -0.38 (-1.76, 0.99) | 0.575 |
|  | Adults and children | 7 | 0.74 (-0.3, 1.78) | 0.158 |
|  |  |  |  |  |
| **Direction of price change** | Decrease (subsidy) | 22 | (reference) | - |
|  | Increase (tax) | 15 | 0.77 (0.02, 1.52) | **0.044** |
|  |  |  |  |  |
| **No. of additional components** | None | 10 | (reference) | - |
|  | 1-2 components | 27 | -0.73 (-1.55, 0.1) | 0.083 |
|  |  |  |  |  |
| **Type of additional components ^3^** | None | 10 | (reference) | - |
|  | Food availability | 6 | -0.68 (-1.86, 0.49) | 0.244 |
|  | Labeling | 11 | 0.2 (-0.24, 0.65) | 0.361 |
|  | Nutritional education | 9 | 0.12 (-0.45, 0.69) | 0.676 |
|  | Food promotion | 11 | -0.1 (-0.61, 0.41) | 0.701 |
|  |  |  |  |  |
| **Quality score ^4^** | 2-3 points | 21 | (reference) | - |
|  | 4-5 points | 16 | 0.80 (0.06, 1.54) | **0.034** |
|  |  |  |  |  |

^1^For each different strata, the values represent the additional elasticity (percentage change in consumption for each percentage change in price) compared to the reference group, based on meta-regression. Compared to the overall findings, negative values represent greater elasticity: i.e., a greater inverse relationship between price and consumption; while positive values represent lower elasticity. For example, studies with price increases found lower overall elasticity, compared to studies with price decreases. Findings are based on 37 intervention arms across 30 studies.

^2^Evaluated in post-hoc analyses after we reviewed the types of settings evaluated in these studies. Too few studies were performed in restaurants (n=1) or schools (n=1) to include these settings in the meta-regression.

^3^Some studies included more than 1 additional component.

^4^Based on 5 criteria: study design, assessment of exposure, assessment of outcome, control for confounding, and evidence of selection bias (see Supplemental Table 1). For each criterion, each study received a score of 1 or 0 (1 being better), and an overall quality score was calculated as the sum of individual scores. We had anticipated performing meta-regression using the continuous score, based on the theoretical range of 0 to ^5^In practice, one study had score=2, one study had score=5, and the remainder had scores of either 3 or 4.
